# Genome‐wide association study identifies an NLR gene that confers partial resistance to *Magnaporthe oryzae* in rice

**DOI:** 10.1111/pbi.13300

**Published:** 2019-12-15

**Authors:** Ming‐Hao Liu, Houxiang Kang, Yucheng Xu, Ye Peng, Dan Wang, Lijun Gao, Xuli Wang, Yuese Ning, Jun Wu, Wende Liu, Chengyun Li, Bin Liu, Guo‐Liang Wang

**Affiliations:** ^1^ State Key Laboratory for Biology of Plant Diseases and Insect Pests Institute of Plant Protection Chinese Academy of Agricultural Sciences Beijing China; ^2^ Hunan Provincial Key Laboratory of Crop Germplasm Innovation and Utilization and College of Agronomy Hunan Agricultural University Changsha Hunan China; ^3^ Guangxi Crop Genetic Improvement and Biotechnology Laboratory Guangxi Academy of Agricultural Sciences Nanning China; ^4^ State Key Laboratory of Hybrid Rice Hunan Hybrid Rice Research Centre Changsha Hunan China; ^5^ The Ministry of Education Key Laboratory for Agricultural Biodiversity and Pest Management Yunnan Agricultural University Kunming China; ^6^ Guangdong Key Laboratory of New Technology in Rice Breeding Rice Research Institute Guangdong Academy of Agricultural Sciences Guangzhou China; ^7^ Department of Plant Pathology Ohio State University Columbus OH USA

**Keywords:** rice blast, *Magnaporthe oryzae*, genome‐wide association study, partial resistance gene, pathogenic diversification, *PiPR1*

## Abstract

Because of the frequent breakdown of major resistance (*R*) genes, identification of new partial *R* genes against rice blast disease is an important goal of rice breeding. In this study, we used a core collection of the Rice Diversity Panel II (C‐RDP‐II), which contains 584 rice accessions and are genotyped with 700 000 single‐nucleotide polymorphism (SNP) markers. The C‐RDP‐II accessions were inoculated with three blast strains collected from different rice‐growing regions in China. Genome‐wide association study identified 27 loci associated with rice blast resistance (LABRs). Among them, 22 LABRs were not associated with any known blast *R* genes or QTLs. Interestingly, a nucleotide‐binding site leucine‐rich repeat (NLR) gene cluster exists in the LABR12 region on chromosome 4. One of the NLR genes is highly conserved in multiple partially resistant rice cultivars, and its expression is significantly up‐regulated at the early stages of rice blast infection. Knockout of this gene via CRISPR‐Cas9 in transgenic plants partially reduced blast resistance to four blast strains. The identification of this new non‐strain specific partial *R* gene, tentatively named rice blast *Partial Resistance gene 1* (*PiPR1*), provides genetic material that will be useful for understanding the partial resistance mechanism and for breeding durably resistant cultivars against blast disease of rice.

## Introduction

Rice feeds more than half of world’s population (Han and Huang, 2013; Leung, 1988), but global rice production is seriously threatened by rice blast disease, which is caused by the fungus *Magnaporthe oryzae* (Dean *et al.*, 2012; Ma *et al.*, 2015). Due to host shift, *M. oryzae* now also threatens wheat production in South America and South Asia (Inoue *et al.*, 2017). Cultivation of resistant varieties is the most effective way to reduce rice yield losses caused by blast. Although> 80 blast resistance (*R*) genes and hundreds of QTLs have been identified (Kang *et al.*, 2016), the fungus overcomes the resistance resulting from a single *R* gene within few years of cultivation of large fields (Xu *et al.*, 2017). Combining *R* genes with QTLs or partial *R* genes (i.e. genes that provide resistance that is not race specific) contributes to broad spectrum and durable resistance to *M. oryzae* (Nelson *et al.*, 2018; Wang *et al.*, 1994). Therefore, identifying additional partial *R* genes in the rice germplasm is important for breeding rice with blast resistance.

Genome‐wide association study (GWAS) has been widely used for genetic mapping and for dissecting the genetic architecture of many agronomic traits in plants (Burghardt *et al.*, 2017). GWAS has also been successfully used for mapping disease resistance loci in crops. For instance, researchers used GWAS to identify 97 loci associated with resistance to stripe rust in wheat (Maccaferri *et al.*, 2015) and 116 loci associated with resistance to corn borers, head smut and dwarf disease in maize(Liu *et al.*, 2014; Samayoa *et al.*, 2015; Wang *et al.*, 2012). In rice, 19 bacterial blight resistance loci (Dilla‐Ermita *et al.*, 2017; Zhang *et al.*, 2017) and about 60 loci associated with indica‐specific resistance to rice blast have been reported (Wang *et al.*, 2014). Using the Rice Diversity Panel‐I (RDP‐I), two recent studies identified> 100 loci associated with rice blast resistance (Kang *et al.*, 2016; Zhu *et al.*, 2016). Although these studies did identify candidate genes, in most cases they did not clone new *R* genes using genetic methods.

The majority of the cloned plant *R* genes encode NLR proteins that result in complete resistance (Li *et al.*, 2015), and only a few genes encode NLR proteins that confer partial resistance to pathogens. For instance, the *Solanum microdontum* gene *R_Pi‐mcd1_* confers partial resistance to *Phytophthora infestans* and belongs to the NLR family (Tan *et al.*, 2008)_._ Another example is the nematode resistance gene *Hero*, which confers a high level of resistance to one species of potato cyst nematode, *Globodera rostochiensis* and partial resistance to another species of potato cyst nematode, *G. pallida* (Ernst *et al.*, 2002). In rice, both *Pi35* and *Pb1* are NLR genes that confer partial resistance to rice blast (Hayashi *et al.*, 2010; Nguyen *et al.*, 2006).

In this study, we used a core collection (584 accessions) of the Rice Diversity Panel II (C‐RDP‐II) to evaluate resistance against three *M. oryzae* strains. The C‐RDP‐II accessions were previously genotyped with 700 000 SNPs (McCouch *et al.*, 2016). GWAS between the rice blast phenotypes and the high‐density SNP genotypes enabled us to identify 27 loci associated with blast resistance (LABR). More importantly, we identified an NLR gene in LABR12 that is induced during *M. oryzae* infection. The disease phenotypic analysis of the CRISPR‐Cas9 mutant of the gene indicated that it functions as a partial resistance gene, and we therefore named it rice blast *Partial Resistance gene 1* or *PiPR1*. The identification of the NLR partial *R* gene in this study provides unique genetic material that can be used to increase our understanding of rice immunity and that will facilitate the breeding of rice cultivars with durable resistance to *M. oryzae*.

## Results

### Rice blast evaluation of C‐RDP‐II

**Figure 1:**
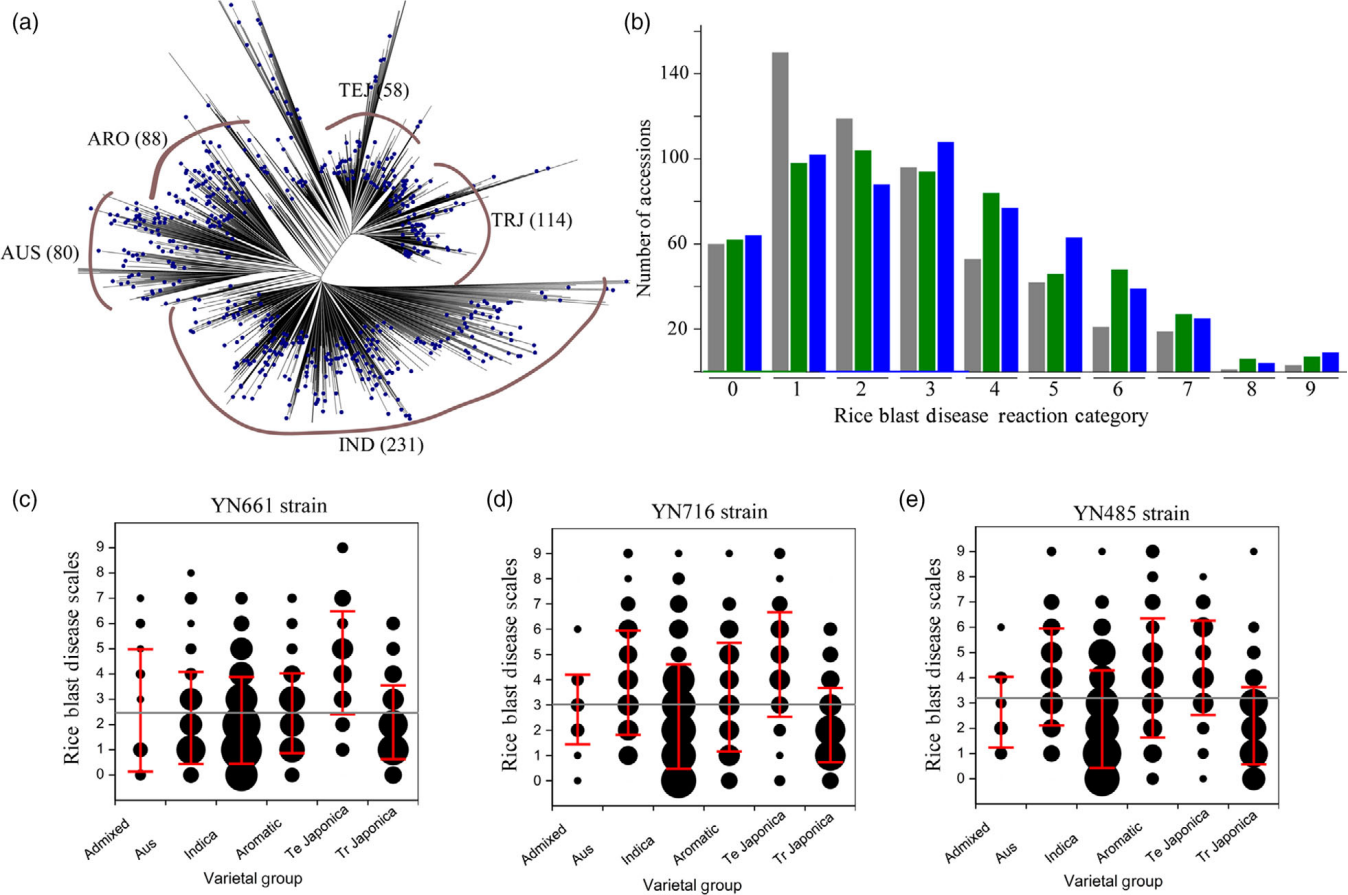
Phylogenetic analysis of the C‐RDP‐II population and evaluation of rice blast resistance of the accessions with three *M. oryzae *strains. (a) Phylogenetic tree of the 584 accessions in the C‐RDP‐II population. (b) Distribution of the rice blast evaluation results of the C‐RDP‐II accessions following inoculation with YN661 (grey), YN716, (green) or YN485 (blue). The *x*‐axis indicates the 0–9 rice blast reaction category, and the *y*‐axis indicates the number of rice accessions. Blast resistance scores of six rice sub‐populations to YN661(c), YN716 (d) and YN485 (e). The *x*‐axis indicates the different sub‐populations, and the *y*‐axis indicates the 0–9 rice blast scores. The size of the black circle represents the number of rice accessions.

Among the 584 accessions of the C‐RDP‐II population, 231 are indica (IND), 114 are tropical japonica (TRJ), 58 are temperate japonica (TEJ), 88 are aroma (ARO), 80 are aus (AUS) and 13 are area mixture (ADM). As shown in Figure 1a, a phylogenetic tree was constructed using the 700 000 SNPs in the 584 accessions previously generated from the RDP‐II population (McCouch *et al.*, 2016). To evaluate the rice blast resistance phenotypes of the C‐RDP‐II population, we selected three representative strains of *M. oryzae* (YN661, YN716 and YN485), which were collected from different rice cultivation regions in Yunnan Province, China (Saleh *et al.*, 2014). YN661, YN716 and YN485 were isolated from a temperate japonica cultivar, an indica cultivar, and an indica cultivar in a region with mixed plantings of indica and japonica cultivars, respectively. All of the accessions in C‐RDP‐II were inoculated with the three strains and then evaluated for rice blast on a 0–9 scale: scores 0–3 were classified as resistant (R), and scores 4–9 were classified as susceptible (S) (Kang *et al.*, 2016). Blast infection data were obtained with at least three replications from 564, 576 and 578 accessions for strains YN661, YN716 and YN485, respectively (Table S1; Figure 1b). The percentage of the accessions susceptible to YN661, YN716 and YN485 was 32.11%, 44.18% and 54.11%, respectively, indicating that YN485 from the indica and japonica mixed region was more virulent than the other two strains.

Among the inoculated accessions, 60 were highly resistant to all three strains (scores 0–1; Table S2). Among these highly resistant accessions, 38, 17, 3 and 2 belonged to the sub‐populations of IND, TRJ, ARO and AUS, respectively, and no highly resistant accession was identified in TEJ and ADM. Analysis of their origins indicated that half of the highly resistant accessions were collected from the Philippines (8 accessions), India (6 accessions), Bangladesh (4 accessions), China (3 accessions), Brazil (3 accessions), Ivory Coast (3 accessions) and Sri Lanka (3 accessions). Among the highly resistant accessions, only one (121339, Farox 299) had been previously reported to be resistant to rice blast (Awoderu, 2010).

### Resistance of the sub‐populations to the three *M. oryzae* strains

Next, we compared the resistance of the five major sub‐populations (IND, TRJ, TEJ, ARO, ADM and AUS) to the three strains. For YN661, the percentage of susceptible accession in IND, TEJ, TRJ, ARO and AUS(ADM was not included due to few accessions) was 27.51%, 76.79%, 25.47%, 33.33% and 21.25%, respectively, (Figure1c); for YN716, the percentage of susceptible accessions was 35.93%, 84.48%, 27.19%, 48.86% and 57.50%, respectively (Figure 1d); and for YN485, the percentage of susceptible accessions was 38.53%, 84.48%, 34.21%, 69.32% and 88.75%, respectively (Figure 1e). These results indicate that TEJ was the most susceptible sub‐population and that TRJ was the most resistant.

### Identification of LABRs and their haplotypes

**Figure 2:**
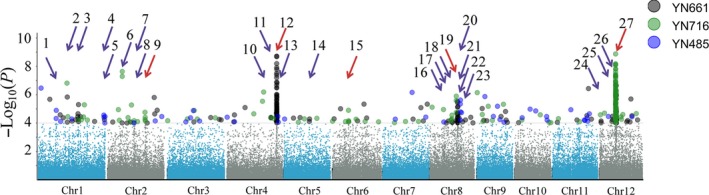
Genome‐wide association analysis of disease resistance using C‐RDP‐II and three *M. oryzae* strains. The Manhattan plots of SNPs on 12 rice chromosomes. The *x*‐axis indicates the genomic coordinates, and the *y‐axis* indicates the association score of each SNP; the score represents a transformed *P* value, ‐log_10_ *P*. The red arrows indicate the loci that are co‐localized with previously mapped or cloned rice blast resistance genes, and the purple arrows indicate the novel loci identified in this study.

Using the 700,000 SNPs and the rice blast resistance phenotypes of the C‐RDP‐II accessions, we identified 27 LABRs (LOD ≥ 4.0). LABR12 and 27 had the highest –LOD scores (8.71 and 8.87, respectively). LABR12 is located at the terminal of chromosome 4, is co‐localized with a previously identified *R* locus (Kang *et al.*, 2016) and the *R* gene *Pi63* (Xu *et al.*, 2014). In the reference genome of Nipponbare (NPB), six NLR genes (*Os04g52970*, *Os04g53000*, *Os04g53030*, *Os04g53050*, *Os04g53120* and *Os04g53160*) form a tandem‐repeat *R*‐gene cluster in the LABR12 region. LABR27 is co‐localized with two cloned *R* genes, *Pita* (Bryan *et al.*, 2000) and *Ptr *(Zhao *et al.*, 2018), and a previously identified *R* locus (Kang *et al.*, 2016). Among the 27 LABRs, 5 are co‐localized with previously mapped or cloned *R* genes, and the other 22 are new (Table 1, Figure 2). Using the NPB genome (MSU v7.0) sequence as a reference, we analysed the 200‐kb genome sequences around the top‐associated SNPs of the 22 novel LABRs. These regions contain 733 protein‐coding genes. Among them, only 11 typical NLR genes were found at four loci.

**Table 1:** The top 27 LABRs associated with rice blast resistance identified in this study

Locus	Top SNP	Chromosome	Number of SNPs (*P *< 1E‐04）	*P* value (The top SNP)	Allele	MAF (%)	Annotation
LABR1	SNP‐1.12217695	1	5	1.29E‐05	G/A	9.32	
LABR2	SNP‐1.19229869	1	2	1.47E‐06	G/A	4.88	
LABR3	SNP‐1.26263915	1	32	3.22E‐06	T/G	13.69	
LABR4	SNP‐1.42487866	1	2	2.77E‐05	C/T	13.89	
LABR5	SNP‐1.43251167	1	10	3.52E‐05	G/A	8.55	
LABR6	SNP‐2.9233957	2	2	2.32E‐08	C/G	31.03	
LABR7	SNP‐2.23689378	2	3	1.75E‐05	G/A	25.29	
LABR8	SNP‐2.23818053	2	4	3.98E‐05	G/A	20.08	
LABR9	SNP‐2.29578362	2	2	1.58E‐06	A/G	5.70	*LAFBR‐1, LAFBR‐2*
LABR10	SNP‐4.27726859	4	2	3.98E‐05	C/T	32.52	
LABR11	SNP‐4.31159185	4	4	1.99E‐05	C/G	8.07	
LABR12	SNP‐4.31381117	4	111	1.9329E‐09	C/T	29.95	*LABR_44*
LABR13	SNP‐4.32143028	4	3	3.39E‐05	G/A	43.58	
LABR14	SNP‐5.16415687	5	5	5.20E‐05	C/A	13.79	
LABR15	SNP‐6.10282193	6	10	1.27E‐05	G/A	4.42	*Pi‐9,Pi‐2,Pi‐zt,Pi‐40,Pigm, LABR_48*
LABR16	SNP‐8.13675253	8	2	5.89E‐05	G/A	8.58	
LABR17	SNP‐8.14149561	8	2	2.78E‐05	C/T	14.96	
LABR18	SNP‐8.14543854	8	3	8.74E‐05	G/A	23.12	
LABR19	SNP‐8.16900872	8	3	1.28E‐06	G/A	27.99	*LABR_56*
LABR20	SNP‐8.17688794	8	8	2.93E‐06	G/A	12.77	
LABR21	SNP‐8.17751904	8	35	3.67E‐06	C/A	10.39	
LABR22	SNP‐8.17939398	8	5	6.87E‐06	G/A	11.84	
LABR23	SNP‐8.19591832	8	4	2.42E‐06	G/A	3.59	
LABR24	SNP‐12.1111072	12	2	7.26E‐05	T/C	38.66	
LABR25	SNP‐12.8410324	12	2	3.37E‐05	G/A	11.80	
LABR26	SNP‐12.10296137	12	26	8.38E‐09	C/T	34.82	
LABR27	SNP‐12.10346770	12	208	1.35E‐09	T/A	36.55	*Pi‐h‐1(t), Pi‐ta, Pi‐42, LABR_87*

MAF, Minor allele frequency.

We next made a rice blast‐resistant haplotype map using the 27 LABRs identified in this study (Table 1, column six). Using the associated SNPs in those LABRs to represent the loci, a correlation analysis indicated that the 27LABRs can explain 87.4%, 63.2% and 61.0% of the phenotypic variation in resistance to YN661, YN716 and YN485, respectively. Haplotype map identified in this study could be useful for marker‐assisted selection (MAS) in rice molecular breeding.

### Identification of a novel partial resistance gene in LABR12

**Figure 3:**
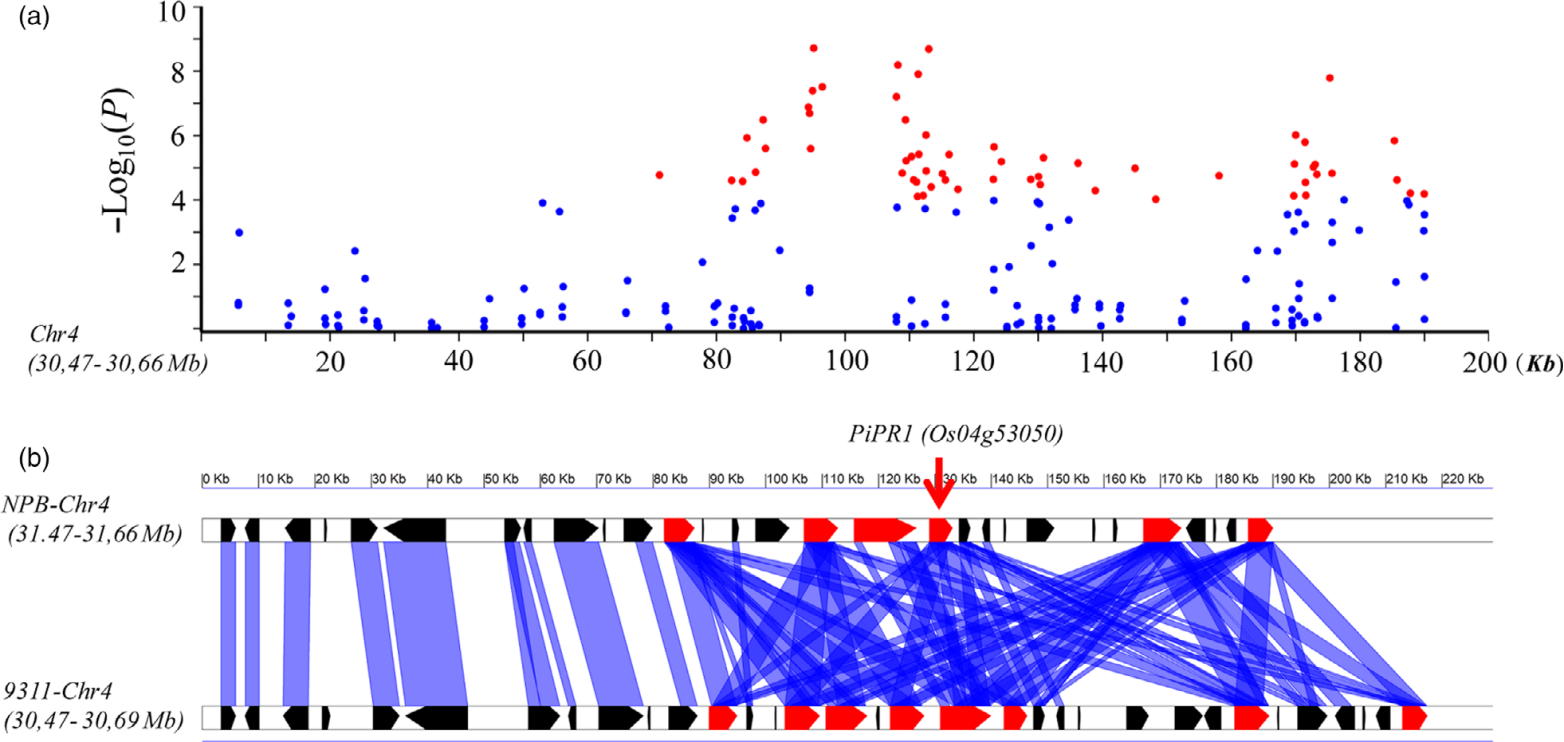
The SNPs and synteny of the genomic sequences of the ~ 200‐kb region at the LABR12 locus. (a) Manhattan plots of SNPs at LABR12. The *x*‐axis indicates the distance (kb), and the *y‐axis* indicates the association score of each SNP. The score represents a transformed *P* value, −log_10_ *P*. The blue and red dots indicate SNPs with a LOD score < 4.0 and ≥ 4.0, respectively. (b) Synteny analysis of the LABR12 locus between the Nipponbare and the 9311 genomes. The blue bars link the homologous genes between the genomes. The red polygons represent the NBS‐LRR‐type *R* genes. The red arrow indicates the *PiPR1* gene(*Os04g53050*).

In the 200‐kb region of LABR12, many significant SNPs were tightly associated with rice blast resistance (Figure 3a). Interestingly, multiple NLR genes are localized in this region and form a tandem‐repeat *R‐*gene cluster. Synteny analysis of the 200‐kb genome sequences of LABR12 detected 8 NLR genes in the indica cultivar 9311 and 6 NLR genes in the japonica cultivar NPB (Figure 3b). The *R* gene *Pi63* is the only previously cloned NLR gene in the region (Xu *et al.*, 2008; Xu *et al.*, 2014). However, the sequence of *Pi63* is not the same as that of any of the NLR genes in the reference genomes of NPB and 9311.

**Figure 4:**
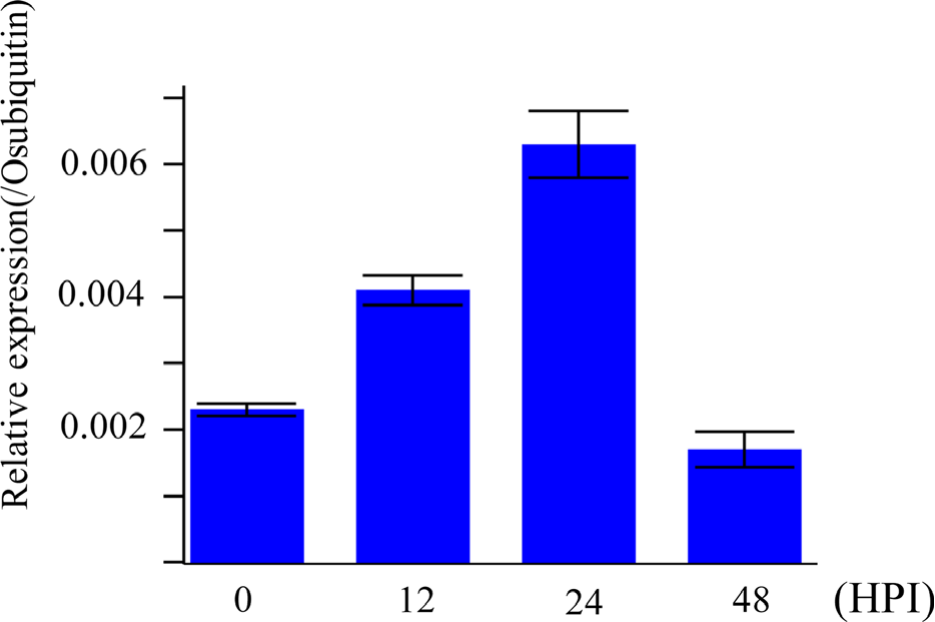
qRT‐PCR analysis of the *PiPR1* gene during *M. oryzae* infection. The *x‐axis* indicates different time points (hour) post inoculation (hpi). The *y*‐axis indicates the relative transcription level of the *PiPR1* gene. The rice housekeeping gene *Ubiquitin* was used as an internal reference.

**Figure 5:**
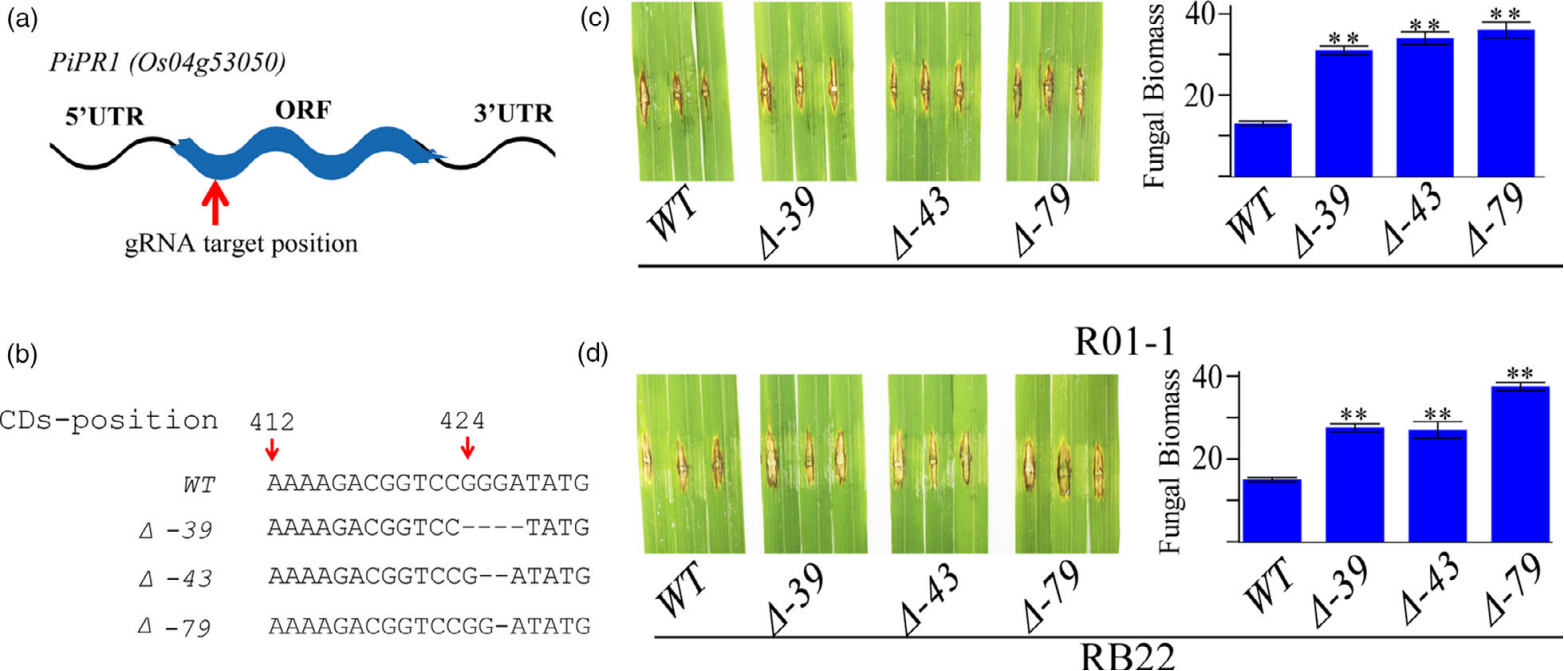
Functional analysis of PiPR1 using the CRISPR‐Cas9 gene editing strategy. (a) Mutations in the three knockout mutants of *PiPR1*in the NPB background. (b) Punch inoculation of the three mutants and wild‐type plants using *M. oryzae* strain R01‐1. Images of inoculated leaves are on the left, and the fungal biomass in the inoculated leaves is indicated on the right. (c) Punch inoculation of the three mutants and wild‐type plants using strain RB22. Images of inoculated leaves are on the left, and the fungal biomass of the inoculated leaves is indicated on the right. WT: wild‐type NPB at the control.

**Figure 6:**
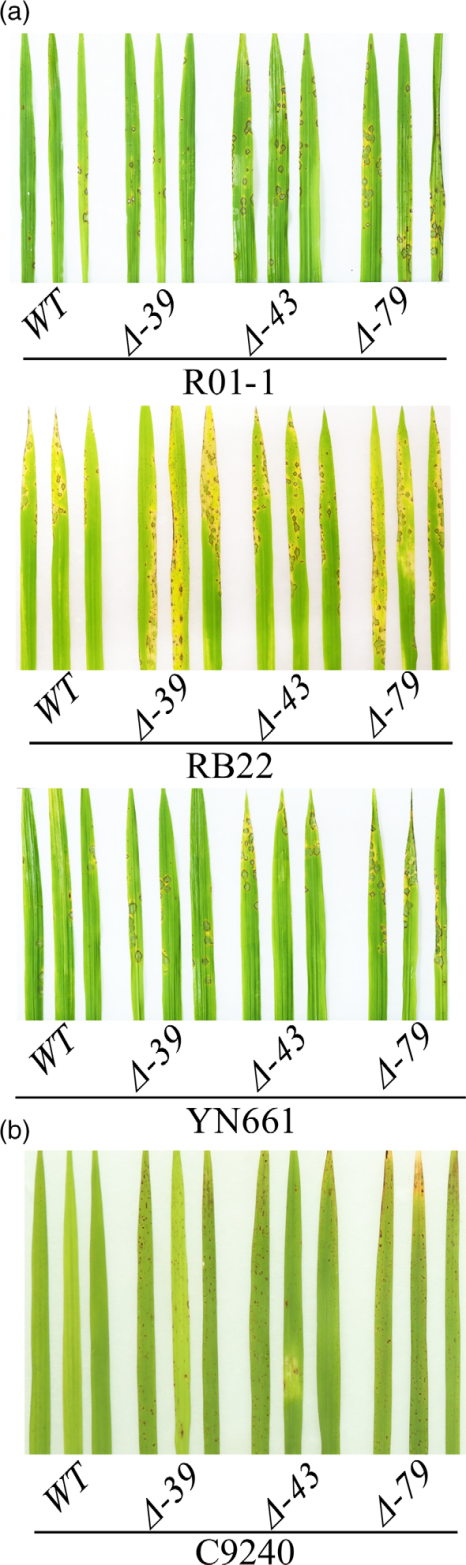
Spray inoculation of the *PiPR1* knockout mutants and wild‐type plants with four *M. orzyae strains*. Spray inoculation of the *PiPR1* mutants and wild‐type plants with (a) three compatible strains (R01‐1, RB22 and YN661) or with (b) an incompatible strain (C9240). WT: wild‐type NPB as the control.

To investigate whether LABR12 resistance is due to *Pi63*, we selected 10 highly resistant and 10 partially resistant accessions in the C‐RPD‐II population for sequencing analysis of the LABR12 locus. Using the NPB and 9311 genomic sequences as well as*Pi63* as references, the LABR12 region in these cultivars was cloned and sequenced. We found that one NLR gene, *Os04g53050*, but not *Pi63*, is highly conserved in multiple rice accessions with partial resistance to *M.oryzae* (Figure 3b).To investigate *Os04g53050* expression during *M.oryzae* infection, we performed quantitative real‐time PCR (qRT‐PCR) using RNA isolated from the inoculated NPB leaves. The analysis showed that *Os04g53050* was significantly up‐regulated in the early stage of infection, that is 12 and 24 hour post inoculation (hpi) (Figure 4), suggesting that *Os04g53050* may be associated with partial resistance to *M. oryzae*. To test that hypothesis, we knocked out this gene in the NPB background with the CRISPR/Cas9 gene editing system (Zhou *et al.*, 2014). We designed one gRNA that is located in the 412 bp of *Os04g53050* (Figure 5a). We obtained eight CRISPR mutants with different mutations in the coding region of *Os04g53050* (Table S3). We then selected the following three T2 homozygous frameshift mutants at the 5′ region of the *Os04g53050* gene for blast inoculation: *PiPR1*‐39, *PiPR1*‐43 and *PiPR1*‐79 (Figure 5b). The results of both punch inoculation (Figure 5c and d) and spray inoculation (Figure 6a) showed that all three knockout mutants were more susceptible than WT to the three *M. oryzae*strains. To further investigate the function of *Os04g53050*, we inoculated the three mutants with the incompatible strain C9240. To our surprise, we observed no lesions on the WT but small lesions on the three knockout mutants (Figure 6b). These results demonstrated that knockout of *Os04g53050* in rice decreases its resistance to both compatible and incompatible *M. oryzae* strains and that *Os04g53050*, which was named *PiPR1*, confers partial resistance to *M. oryzae*.

To determine the natural variation of the *PiPR1* locus in rice cultivars, we analysed the sequence polymorphism of the locus in 122 rice cultivars, which were included in the 3K‐Rice Genome Project (Wang *et al.*, 2018). Surprisingly, the locus was highly polymorphic and 112 genotypes were identified among the 122 rice cultivars (Table S4). Among the identified genotypes, 9 have a premature stop codon, 17 have frameshift mutations, and 17 have both a premature stop codon and frameshift mutations. These results indicate that the *PiPR1*locus is diversified and may have been under rice blast pathogen selection during the co‐evolution between rice and *M. oryzae*.

## Discussion

Consistent with our previous research (Kang *et al.*, 2016; Zhu *et al.*, 2016) using the Rice Diversity Panel I, the current study indicated that the TRJ and IND sub‐populations of rice are more resistant than the TEJ sub‐population even though the current study used different *M. oryzae*strains than the earlier studies. The TEJ rice is the major crop in North China, Japan, and Korea. In recent years, the TEJ rice has been increasingly cultivated in south‐central and western China. The increased cultivation of TEJ cultivars in China is likely to select for *M. oryzae* genotypes that can overcome the resistance in those cultivars, resulting in epidemics of rice blast disease in some TEJ‐growing regions. A recent report indicated that some IND rice varieties are more resistant than TEJ varieties due to presence of the single *R* gene, *Pia* (Liao *et al.*, 2016). Therefore, breeders should introduce effective *R* genes into the TEJ cultivars.

Rice blast *R* genes have been used for several decades due to their strong resistance and their ease of selection in rice breeding (Wang and Valent, 2017). Although some *R* genes such as *Pi9* confer broad‐spectrum and durable resistance, most are overcome by the pathogen within a few years in the field. Partial *R* genes, in contrast, are non‐race specific and are associated with durable resistance (Mundt, 2014). Therefore, identification of partial *R* genes is a priority in rice breeding programmes. Even though substantial effort has been applied to the mapping and cloning of partial *R* genes or QTLs, only three partial *R* genes against *M. oryzae* (*pi21*, *Pi35* and *Pb1*) have been cloned in rice (Fukuoka *et al.*, 2009; Hayashi *et al.*, 2010; Nguyen *et al.*, 2006). In this study, we found that multiple NLR genes are localized at the LABR12 locus where several NLR genes are also clustered. Through bioinformatic and expression analyses, we identified an NLR gene, *PiPR1*, that confers partial resistance to *M. oryzae*. Inoculations of the knockout mutants with three compatible isolates revealed that mutations in *PiPR1* significantly reduce resistance to *M. oryzae*. The *PiPR1* mutants also showed reduced resistance to the incompatible strain C9240. How this locus is evolved during the co‐evolution with *M. oryzae* and functions in rice immunity are not clear. Sequence analysis of the *PiPR1* locus in 122 cultivars showed that the gene is highly diversified with different indel mutations, which are localized in the entire coding region. Whether all these mutations cause the loss of partial resistance to *M. oryzae* warrants further investigation. Furthermore, we found that there is no NLR sensor‐helper gene pair (Wang *et al.*, 2019) at this locus, suggesting that the mechanism of *PiPR1* may be different compared with other rice blast *R* genes. It is noteworthy that *Xa38*, a previously reported bacterial blight resistant locus (Ellur *et al.*, 2018), is co‐localized with the *PiPR1* locus. It will be interesting to check whether the *PiPR1* knockout lines also confer enhanced susceptibility to the bacterial blight pathogen. Nevertheless, identification of this non‐strain‐specific partial *R* gene not only provides important genetic material for rice breeding but also raises several intriguing questions. For example, does *PiPR1* recognize an avirulence effector in *M. oryzae?* Does it activate similar defence signalling pathways as other *R* genes? What is its relationship with other *R* genes? Further investigation of *PiPR1*‐mediated resistance in rice should provide new insights into the mechanism of partial resistance against *M. oryzae*in rice.

## Materials and methods

### Plant materials and blast inoculation

The C‐RDP‐II accessions used in this study were obtained from Guangdong Academy of Agricultural Science. Three‐week‐old seedlings were used for *M. oryzae* inoculations. The *M. oryzae* strains YN485, YN661 and YN716 were originally collected from Yunnan Province.

### Evaluation of rice blast resistance

Blast resistance of the 584 accessions was evaluated as previously described (Mgonja *et al.*, 2016). In brief, disease was scored 6 days after inoculation using a 0–9 blast scale, in which ‘0’ indicated no blast symptoms (highly resistant) and ‘9’ indicated severe blast symptoms (highly susceptible; Zhu *et al.*, 2012). Three biological repeats were used for each rice accession.

### RNA extraction and qRT‐PCR analysis

Rice NPB leaves infected with *M. oryzae* strains were collected at different time points after inoculation. Total RNA was isolated as described previously (Chan *et al.*, 2007) with Trizol reagent (Invitrogen reagent number 15596026) and was then purified with a Promega kit (reagent number A5001). qRT‐PCR was performed using the Real Star Green Fast Mixture (Gene Star reagent number 8AH01). The rice ubiquitin gene was used as an internal control, and the 2^−△△Ct^ method was used to calculate the relative expression level (Livak, 2001). Primer sequences are listed in Table S5.

### Genome‐wide association analysis

We used a similar GWAS method as previously described (Kang *et al.*, 2016; McCouch *et al.*, 2016). In brief, a mixed linear model (MLM) (Bradbury *et al.*, 2007) with integrated kinship (K) and population structure (Q) matrices in Tassel 5.0 software (https://www.maizegenetics.net/tassel) was used. We then constructed the integrated Manhattan plots from GWAS output used Perl scripts based on PERL and its SVG module (scalable vector graphics). The LOD score [log10 (odds ratio)] was calculated as previously described (Kang *et al.*, 2016). It is a statistical test used in genetic linkage analysis. The LOD score compares the probability of obtaining the test data if the two loci are linked to the probability of obtaining the test data if the two loci are not linked.

### Construction of CRISPR‐CAS9 target‐gene mutants

The CRISPR‐Cas9 gene mutation system was obtained from the Zhou laboratory in the Institute of Plant Protection, Beijing China (Zhou *et al.*, 2014). The target primers were designed using the online website (http://skl.scau.edu.cn/primerdesign/) and are listed in Table S5. The CRISPR vectors were transformed into NPB by an agrobacterium‐mediated transformation method (Lu *et al.*, 2007).

## Conflicts of interest

The authors declare no conflicts of interest.

## Author contributions

HK and G‐LW designed and initiated this project and supervised the experiments. ML, HK, YX, DW, LG, XW, YN, JW, WL, CL and BL performed experiments. HK, ML and G‐L W analysed the data. HK, ML and G‐L W composed the manuscript. All authors have discussed the results and commented on the manuscript. All authors have read and approved the final manuscript.

## Supporting information


**Table S1** Blast disease scores of 600 rice accessions (C‐RDP‐II) inoculated with strains YN661, YN716, or YN485 of *M. orzyae*.

**Table S2** List of the rice accessions highly resistant to *M. orzyae*strains YN661, YN716, and YN485.

**Table S3** The eight genotypes obtained from CRISPR‐CAS9 target editing of *PiPR1*gene.

**Table S4** Natural variations of the *PiPR1* locus in the 122 rice cultivars.

**Table S5** Primers used in this study.
